# Wisdom from the past

**Published:** 1971-01

**Authors:** 


### Wisdom from the Past

(Extracts from this Journal 1896 and 1921)

### SEVENTY-FIVE YEARS AGO

**BRISTOL—"Health Report"**—The annual Report of the Medical Officer of Health for 1895 has been recently distributed, and contains much valuable and interesting information on the health of the city and the means which are taken to keep the condition of the health-rate up to its present highly satisfactory state.

Part I. is the "Report of the General Sanitary Condition of the Urban Sanitary District of Bristol". We are glad to find that the *old wells* which still exist are gradually being closed. All of those examined (43) during the year were found to be more or less polluted—a fact we can hardly be surprised at when we remember that the soil has been lived over for centuries. In one case the users had attempted to purify the water by a charcoal filter, with the result of making it fouler than before.

As many of our readers know, the loan (£16,100) for the completion of the *Smallpox Hospital* upon the Novers Hill site (13 acres) has received the sanction of the Local Government Board. This will add 20 beds to the already existing 50, as well as administration, isolation and laundry blocks, and a disinfecting station.

The inquiry into the Ham Green site (20½ acres) was progressing at the time the report was written, the proposed loan being £27,000. The permission has since been granted and the work was begun at once. This will add 76 beds to the present accommodation.

The calculated population of Bristol is now 228,139. During 1895 there was an increase in the *birth-rate,* which had previously been falling off—6,622 births, 29.02 birth rate, compared with 6,393 births, 28.81 birth-rate in 1894; while the *marriage-rate* has fallen off, 10.8 against 11.5 in 1894. *Illegitimate births,* however, have risen to 222 from 189. The number of *deaths* has risen to 4,108 from 3,888 or 18.08 as compared with 17.16—the lowest ever recorded—in 1894. *Infant mortality* has fallen from 948 to 935; the rate is therefore lower—141.19 against 148.31 in 1894. Bristol as usual compared favourably with many big towns. According to the averages, which at best are most deceptive things, 302 persons now living should be dead. We congratulate these individuals, whoever they may be. This net gain is lower than 1894, when it was 550.

As is usually the case, the latest available returns of *vaccination* are those of the previous year (1894), and, as may be easily explained, a considerable falling off is observed, the percentage successfully vaccinated being 71.49 as compared with 76.91. The figures are for the Bristol Union. For the Barton Regis Union the numbers are still lower, 68.6 against 72.0 in 1893.

Owing to the hot summer and autumn, there was a great increase in fatal cases of *diarrhoea,* this being especially marked in the last two quarters of the year, 143 cases in 1895, as compared with 65 in 1894.

*Diphtheria* cases increased slightly, 165 against 129 in 1894 and 142 in 1893; but the mortality appears to have decreased, 20.6 against 39 in 1894.

The bacteriological examination of the cases by Dr. Dowson is interesting. He examined 87 cases, finding the bacillus in 70, and as in the remaining 17 no opportunity was given for a second examination, it is possible they may have been true cases of diphtheria. The medical Officer of Health calls attention to the fact that the examination is to confirm, not to make a diagnosis. We commend to the opponents of enlarging the city boundaries Dr. Dowson's remarks on the incidence rate of diphtheria in the various Bristol districts, especially the following:—"Considering the amount of infection that comes into Bristol from surrounding districts, the present system is rather one of keeping disease within certain limits than of preventing its occurrence".

Only two cases of *smallpox* occurred in the city, but two were received from outside authorities for isolation. We are promised a special report later.

*Erisipelas,* the notification of which the Medical Officer thinks unnecessary, shows a slight increase, as does *scarlet fever,* though there was no epidemic of the latter disease. No case of *typhus* has been known to exist in the city since the 1889-90 winter. The falling-off in the number of *typhoid* cases in satisfactory, due no doubt to the better sanitary conditions in general. Dr. Davies does not think that with our present hospital accommodation the time is ripe for the notification of *measles.* Only 8 deaths occurred from this disease in 1895.

—Vol. 14 pp. 284-285 (1896)

### FIFTY YEARS AGO

Psychotherapy, and especially psycho-analysis, is frequently grossly abused by practitioners who will not distinguish between functional and structural change, and thereby render themselves ridiculous, as for example did the exponent of the art who claimed with some pride at an important medical meeting to have cured a case of epistaxis by eighteen months' psycho-analysis.

The present-day tendency for the laity to practise psycho-analysis is dangerous and pernicious, and the practice of society ladies psycho-analysing their butlers as described in the **Daily Mail** is indecent. On the other hand, many practitioners are blind to the possibilities of psychotherapy, and outside the realm of the psycho-neuroses many conditions commonly regarded as essentially organic can be dealt with by this method. As examples I may mention the cases of pernicious vomiting of pregnancy described by Hurst, in one of which the ammonia coefficient reached 25. All these were ordered by the gynaecologist to have the uterus emptied within twenty-four hours if life was to be saved, and all of them were cured by psychotherapy during that interval and have gone on to full time without further accident. Also two cases of deaf-mutism dating from early infancy who had been cured, one at the age of seventeen and the other at twenty-two.

In medical education the absence of any teaching of medical psychology is a serious deficiency. The course of lectures on mental diseases does not supply this want at all in most schools, and consequently the medical student enters into practice with no knowledge of how to deal with his neurotic patients, which form a very large proportion of his practice, and, what is almost more important, has no organised knowledge of how to approach the subjective symptoms he meets with, which as Sir James Mackenzie has said are really just as important as the objective signs.

—From "The Present Position of Psychotherapy" by R. G. Gordon—Vol. 38, pp. 27-28 (1921).

